# Zerumbone increases oxidative stress in a thiol-dependent ROS-independent manner to increase DNA damage and sensitize colorectal cancer cells to radiation

**DOI:** 10.1002/cam4.367

**Published:** 2014-12-01

**Authors:** Amit Deorukhkar, Niharika Ahuja, Armando-Lopez Mercado, Parmeswaran Diagaradjane, Uma Raju, Nalini Patel, Pranshu Mohindra, Nga Diep, Sushovan Guha, Sunil Krishnan

**Affiliations:** 1 Department of Experimental Radiation Oncology, The University of Texas MD Anderson Cancer CenterHouston, Texas, 77030; 2 Division of Gastroenterology, Hepatology, and Nutrition, The UT Medical School and Health Science Center at Houston6431 Fannin Street, MSB 4.234, Houston, Texas, 77030

**Keywords:** Colorectal cancer, DNA repair, glutathione, radiation, sesquiterpene

## Abstract

Locally advanced rectal cancers are treated with neoadjuvant chemoradiation therapy followed by surgery. In a minority (∽20%) of patients, no tumor is present at the time of surgery; these patients with a complete pathologic response (pathCR) to neoadjuvant therapy have better treatment outcomes. Unfortunately, the inherent radioresistance of colorectal cancer (CRC) cells dictates that the majority of patients do not achieve a pathCR. Efforts to improve these odds have fueled the search for novel, relatively less-toxic radiosensitizers with distinct molecular mechanism(s) and broad-spectrum anticancer activities. Here, we use zerumbone, a sesquiterpene from the edible ginger (*Zingiber zerumbet* Smith), to enhance radiosensitivity of CRC cells. Short exposure to zerumbone (7 h) profoundly sensitized CRC cells, independent of their p53 or *k*-*RAS* status. Zerumbone enhanced radiation-induced cell cycle arrest (G2/M), increased radiation-induced apoptosis, but induced little apoptosis by itself. Zerumbone significantly enhanced radiation-induced DNA damage, as evident by delayed resolution of post-irradiation nuclear *γ*H2AX foci, whereas zerumbone treatment alone did not induce *γ*H2AX foci formation. Zerumbone pretreatment inhibited radiation-induced nuclear expression of DNA repair proteins ataxia-telangiectasia mutated (ATM) and DNA-PKcs. Interestingly, zerumbone-mediated radiosensitization did not involve reactive oxygen species (ROS), but was mediated through depletion of cellular glutathione (GSH). Ability of only thiol-based antioxidants to abrogate zerumbone-mediated radiosensitization further corroborated this hypothesis. The *α*,*β*-unsaturated carbonyl group in zerumbone was found to be essential for its bioactivity as zerumbone analog *α*-Humulene that lacks this functional group, could neither radiosensitize CRC cells, nor deplete cellular GSH. Our studies elucidate novel mechanism(s) of zerumbone's ability to enhance CRC radiosensitivity.

## Introduction

Colorectal cancer (CRC) is the third-leading cause of cancer-related deaths in the US.^1^ For resectable locally advanced rectal cancer, chemoradiation therapy followed by surgical resection is the standard of care. Several randomized trials have studied the impact of dose modification and pre- and postoperative administration of radiation in order to enhance local-regional control of disease.^2^–^4^ All these investigations have unanimously established the unprecedented advantages of preoperative chemoradiotherapy (CRT) over surgery alone as a means to (1) downstage the tumor, (2) make it more resectable, (3) minimize the incidence of postsurgical local recurrence, (4) improve the chances of anal sphincter preservation at the time of surgery, and (5) significantly improve disease-free survival.^2^,^4^–^9^ Furthermore, pathological complete response (pathCR; no tumor left behind at surgery) confers the best clinical outcomes and permits selected patients to undergo less-extensive resections, minimizing the adverse effects of treatment.^5^,^10^ Unfortunately, given the inherent radioresistance of CRC, only about 20% of patients achieve pathCR.^11^ Efforts to overcome the resistance of CRCs to radiation therapy (RT) have largely included intensifying the radiation dose or using radiosensitizing cytotoxic chemotherapy, both of which are associated with increased toxicity.^11^–^14^ The recognition of the clinical significance of CRT and the biological importance of a multi-targeted approach has fueled the quest for newer radiosensitizing agents with broad-spectrum anticancer activities and less toxicity, which can help overcome the intrinsic radioresistance of rectal cancers.

Intrinsic tumor cell radiosensitivity is governed by a plethora of factors, where a complex interplay of nuclear and cytoplasmic signaling cascades collectively decides the fate of an irradiated cell. However, ionizing radiation (IR)-induced DNA double-strand breaks (DSB) (direct DNA damage) and production of reactive oxygen species (ROS) which in turn cause DNA DSBs (indirect DNA damage) are widely acknowledged as principal determinants of radiation-induced cell killing. In defense, cellular antioxidants such as the tripeptide thiol glutathione (GSH), play an essential role in protecting cells against the free radical-induced oxidative stress.^15^ Additionally, cells are equipped with multiple pathways to effectively repair the damaged DNA, and hence, cellular GSH pool and the cell's ability to effectively repair the radiation-induced DNA DSBs are crucial factors governing tumor radiosensitivity.^16^,^17^ Conversely, agents that could deplete cellular GSH or inhibit the DNA damage response (DDR) signaling cascades are potential radiosensitizers.^17^–^19^

Zerumbone is a cyclic sesquiterpene from the rhizomes of the edible ginger plant (*Zingiber zerumbet* Smith), typically found in southeast Asia.^20^ The rhizomes of this plant have been in use as a traditional folk medicine for pain (anti-inflammatory) and as a flavoring agent in cooking.^21^ However, recent studies have shown zerumbone to possess unique and potent anticancer, anti-inflammatory and antiproliferative activities against many cancer types.^22^ Particularly in CRC cells, zerumbone has been shown to inhibit the proliferation of human colonic adenocarcinoma cells, with minimal toxicity toward normal human dermal and colonic fibroblasts.^21^ In a mouse colon carcinogenesis model, dietary zerumbone significantly inhibited the multiplicity of colon adenocarcinomas and suppressed colonic inflammation.^23^ Recently, zerumbone was shown to upregulate the tumor necrosis factor-related apoptosis-inducing ligand (TRAIL) death receptors (DR) 4 and DR5 and potentiate TRAIL-induced apoptosis in human CRC cells.^24^ Taken together, these studies highlight the potent chemopreventive and anti-inflammatory activities of zerumbone. Nevertheless, there is very little evidence whether zerumbone can modulate the effects of cancer therapeutic modalities such as RT and/or chemotherapy.

In the present study, we investigated the role of zerumbone in modulating the radioresponse of CRC in vitro. Dissecting the underlying molecular mechanism of action revealed that zerumbone enhanced radiation-induced cell cycle arrest in G2/M phase and also increased the radiation-induced apoptosis. Zerumbone also significantly delayed the post-IR DNA DSB repair, as evident by prolonged expression of nuclear *γ*H2AX foci. Zerumbone-mediated radiosensitization was mediated by zerumbone's ability to deplete cellular GSH levels. Interestingly, zerumbone treatment neither generated ROS by itself, nor enhanced the radiation-induced ROS generation. Finally, the *α*,*β*-unsaturated carbonyl group was found to be the key structural moiety responsible for zerumbone's bioactivities as *α*-Humulene (HUM), a structural analog of zerumbone lacking this functional group failed to show any toxicity or radiosensitizing effects toward CRC cells. HUM was also unable to deplete cellular GSH, which indicated that GSH depletion was a prerequisite for zerumbone-mediated radiosensitization.

## Material and Methods

### Cell lines and cell culture

Human CRC cell lines were kindly provided by Dr. Ray Meyn (MD Anderson Cancer Center). HCT116 cells were maintained in DMEM/F12 (50:50), HT29 in McCoy's 5A medium, and SW620 cells were cultured in L-15 (Lebovitz) medium. All media were procured from Corning Cellgro® (Mediatech Inc., Manassas, VA) and were supplemented with 10% fetal bovine serum (Sigma Aldrich, St. Louis, MO), 1% Penicillin-Streptomycin, and 2 mmol/L l-glutamine (Life Technologies, Grand Island, NY). All cell lines were authenticated by short tandem repeat (STR) profiling by MD Anderson's Characterized Cell Line Core Facility.

### Chemicals

Zerumbone (Kingherbs Inc., NY) was a kind gift from Dr. Bharat Aggarwal (Department of Experimental Therapeutics, MD Anderson Cancer Center). Millimolar (mmol/L) stock solutions of zerumbone in dimethyl sulfoxide (DMSO) were stored at −20°C. For every experiment, the appropriate zerumbone stock was diluted 1:1000 in culture medium immediately prior to use to obtain the respective micromolar (*μ*mol/L) concentration. The control groups received DMSO diluted accordingly (Final DMSO concentration was 0.1% in all groups). All other fine chemicals were procured from Sigma-Aldrich unless otherwise specified.

### Cell viability assay

The effect of zerumbone on tumor cell viability was assessed by using the XTT: (sodium 3′-[1-(phenylaminocarbonyl)- 3,4-tetrazolium]-bis (4-methoxy-6-nitro) benzene sulfonic acid hydrate) cell proliferation kit (Roche Applied Science, Indianapolis, IN) as described previously.^25^ Briefly, cells (3 × 10^4^/mL) were seeded in 96-well plates and grown overnight. The next day, the medium was aspirated and cells were exposed to different concentrations of zerumbone for 7 h. Next, the zerumbone was aspirated and the wells were rinsed and replenished with fresh medium. After a further 48 h of culture, XTT labeling mixture was added to the cells and incubated for another 4 h. The resulting formazan product was then spectrophotometrically quantified (490 nm) by using an enzyme-linked immunosorbent assay (ELISA) plate reader (Perkin Elmer, Waltham, MA). Results are expressed as percent cell viability for each concentration of zerumbone with respect to untreated controls (0.1% DMSO in medium).

### Clonogenic cell survival assay

Cells were treated with vehicle control (DMSO) or with 5, 10, and 25 *μ*mol/L zerumbone for 4 h and then irradiated with a ^137^Cs unit (4.5 × 5.3 cm) at room temperature (Dose rate 2.8 Gy/min). Following 3 h post-IR, zerumbone was washed, cells were trypsinized and specific cell densities were replated in six well plates to be incubated for colony formation for 10–14 days. Colonies were stained with 0.5% alcoholic crystal violet and then counted (GelCount™, Oxford Optronix Ltd., Abingdon, UK). The fraction surviving a given treatment was calculated with respect to the survival of unirradiated controls (cells treated with DMSO or zerumbone alone). For clonogenic assays involving the antioxidants *N*-Acetyl-l-cysteine (NAC), l-Glutathione reduced and (±)-6-Hydroxy-2,5,7,8-tetramethylchromane-2-carboxylic acid (Trolox manufacturer: Sigma Aldrich, St Louis, MO), cells were pretreated with the respective antioxidants and then coexposed with 25 *μ*mol/L zerumbone. Clonogenic assays were performed in the same way as for zerumbone with radiation. For all clonogenic assays, the radiation dose enhancement factor (DEF) was calculated as the dose (Gy) for radiation alone divided by the dose (Gy) for radiation plus drug (normalized for drug toxicity) for a surviving fraction (SF) of 0.1 or 0.25.

### Flow cytometry

Zerumbone-induced apoptosis was determined by flow cytometry using propidium iodide (PI) staining and immunoblotting of apoptotic proteins. For flow cytometry, cells (1 × 10^5^/mL) were seeded in 100 mm dishes and allowed to attach overnight. The next day, the cells were treated with different concentrations of zerumbone for 7 h. For samples treated with both zerumbone and radiation, cells were irradiated at respective doses of radiation after 4 h of zerumbone addition, and were incubated with zerumbone for further 3 h post-IR. At the end of total 7 h of zerumbone treatment, the drug was washed off, and cells were incubated in drug-free media for further 24 h (for cell cycle analysis) or 48 h (for estimating percent apoptotic cells). At either time points, cells were collected by trypsinization, washed with phosphate-buffered saline (PBS) (2×), and fixed in 70% ethanol overnight (−20°C). Cells were then washed with excess of PBS and treated with Ribonuclease A (5 *μ*g/mL final conc.; 30 min at 37°C). After final wash with PBS, cells were resuspended in PI solution (50 *μ*g/mL PI, 0.1% sodium citrate, 0.1% Triton™ X 100) and after 30 min, were acquired by flow cytometry (Beckman Coulter Altra, Beckman Coulter, Fullerton, CA) and the percent apoptotic cells and cell cycle phase distribution were analyzed by ModFit LT™software (Verity Software House, Topsham, ME).

### Immunoblotting

Protein expression in the whole cell lysates or nuclear extracts was performed by western blotting. For whole cell lysates, cells were lysed in buffer (100 mmol/L HEPES: 4-(2-Hydroxyethyl)piperazine-1-ethanesulfonic acid [pH 7.9], 0.5 mmol/L NaCl, 1 mmol/L EGTA, 3 mmol/L EDTA [pH 8.0] and 1% NP40) supplemented with protease and phosphatase inhibitor cocktails (Roche Applied Science). Nuclear extracts were prepared as described previously.^26^ Lysates (25–50 *μ*g) were fractionated by SDS-PAGE, the proteins were electrotransferred to nitrocellulose membranes and probed with antibodies against the following: Ku70 and poly-(ADP-ribose)-polymerase (PARP) (Santa Cruz Biotechnology, Santa Cruz, CA), ATM (ataxia-telangiectasia mutated), phospho-ATM^Ser1981^, caspase-3, caspase-9 (Cell Signaling technology, Danvers, MA), DNA-dependent protein kinase-catalytic subunit (DNA-PKcs; BD Biosciences, San Jose, CA) and Ku86, *β* actin (Sigma-Aldrich). The blots were next probed with appropriate horseradish peroxidase-conjugated secondary antibodies (Santa Cruz Biotechnology) and developed using ECL™ (GE Healthcare, Piscataway, NJ).

### Immunofluorescence

HCT116 cells grown on 22 × 22 mm coverslips (Corning, NY), were pretreated with 25 *μ*mol/L zerumbone for 4 h and then were irradiated (2 Gy). Samples were processed for phosphorylated histone 2AX (*γ*-H2AX) immunostaining at different time points post irradiation as described previously.^27^ Briefly, cells were washed with PBS, fixed with 1% paraformaldehyde (15 min) and 70% ethanol (15 min) at room temperature. Cells were then treated with 1% NP-40 (30 min), blocked with 5% bovine serum albumin (BSA; 30 min), and incubated with anti- *γ*-H2AX antibody (Millipore, Billerica, MA) in 5% BSA for 2 h. Subsequently, cells were washed with PBS, labeled with Alexa-Fluor® 488-conjugated secondary antibody (Life Technologies) for 30 min and counterstained with 4′,6′-diamino-2-phenylindole (DAPI; 1 *μ*g/mL in PBS) for 5 min. Coverslips were mounted with ProLong gold antifade agent (Life Technologies), examined under fluorescent microscope (Leica, Bannockburn, IL) and images were captured. Nuclear *γ*-H2AX foci were then counted manually from at least 50 cells for each treatment condition by an investigator blinded to treatment conditions.

### ROS assay

Generation of intracellular ROS was measured using cell permeant, fluorogenic CellROX® green reagent (Life Technologies) according to the manufacturer's instructions. Briefly, cells (4 × 10^5^/mL) were seeded in 96 well plates and allowed to grow for 48 h. The medium was aspirated and cells were treated with different concentrations of zerumbone for 4 h, and irradiated at indicated doses of radiation (2 and 4 Gy). Following 30 min post-IR, medium was gently aspirated, and CellROX® green reagent (final concentration 5 *μ*mol/L in medium) was added to each well and the cells were incubated at 37°C for 30 min. Finally, cells were washed with PBS (3×), and PBS was added to each well before analyzing the plate on a multi-mode fluorescence plate reader (BioTek, Winooski, VT; Ex/Em 485/528 nm). The mean signal intensity (in relative fluorescence units [RFU]) for each sample (triplicates) was calculated and averaged.

### Cellular thiol detection

Cellular levels of reduced GSH were estimated using GSH detection reagent ThiolTracker™ Violet (Life Technologies) as per manufacturer's instructions. Briefly, cells (4 × 10^5^/mL) were seeded in 96 well plates and allowed to grow for 48 h. The medium was then aspirated and cells were treated with different concentrations of zerumbone for 4 h. Following drug exposure, the medium was gently aspirated, cells washed with PBS (1×), and prewarmed ThiolTracker™ Violet dye working solution in PBS was added to cells (100 *μ*L/well). After further 30 min of incubation at 37°C, ThiolTracker™ solution was aspirated, cells washed with PBS (1×), and fresh PBS was added to each well. Cells were then analyzed on a multi-mode fluorescence plate reader (BioTek, Winooski, VT; Ex/Em 404/528 nm). The mean signal intensity (RFU) for each sample (triplicates) was calculated and averaged, and the fold change in mean signal intensity was normalized with the respective untreated controls for each cell.

### Statistical analyses

Clonogenic assays were performed in sextuplicates. XTT assays were performed in quadruplicates. All other experiments were performed at least in triplicates. Mean ± SEM of data pooled from three independent experiments is shown unless otherwise specified. The differences between groups were analyzed by paired Student's *t*-test (for two groups) or by one-way ANOVA (for >two groups) as applicable (GraphPad Prism 6.01). For clonogenic cell survival assays, the differences between groups were analyzed by Student's *t*-test for each data set points (2, 4, and 6 Gy). A value of *P* < 0.05 was deemed statistically significant.

## Results

### Zerumbone inhibited the proliferation of CRC cells

**Figure 1:**
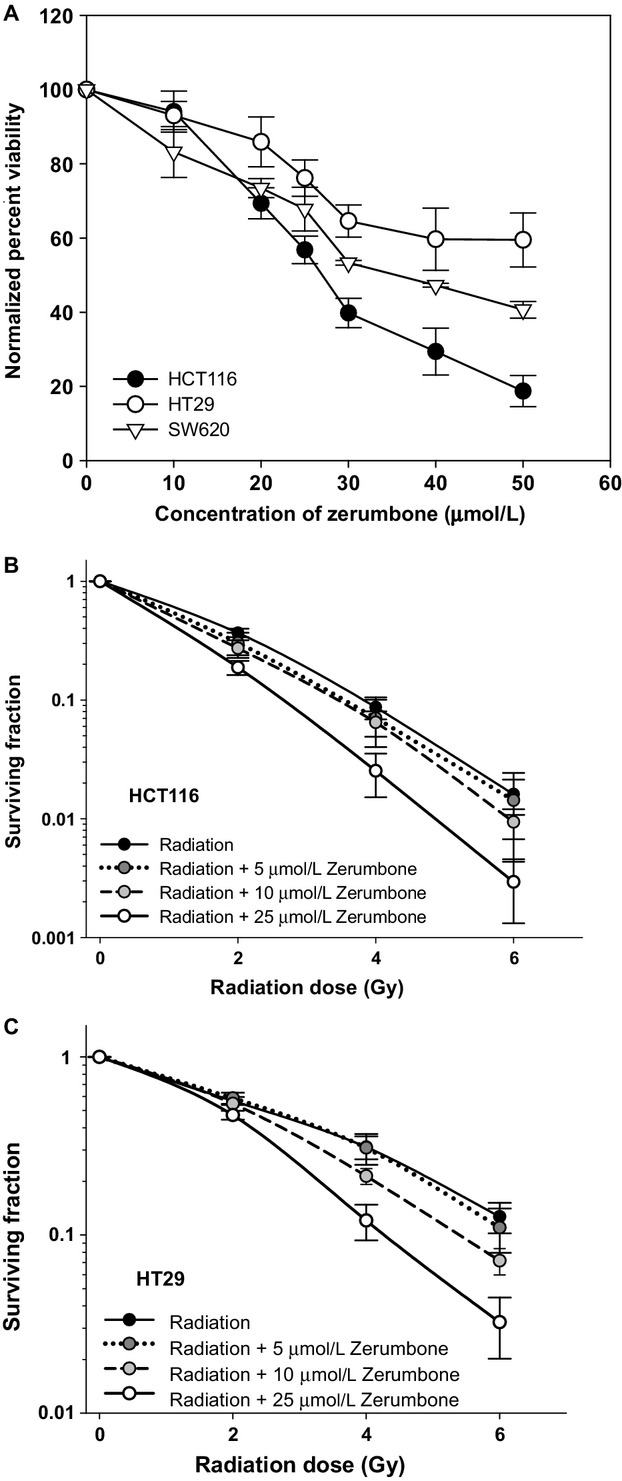
Standalone toxicity of zerumbone and its radiosensitization activity toward colon cancer cells: (A) XTT: Cells (3 × 10^4^/mL) were exposed to different doses of zerumbone in 96-well plate for 7 h after which the zerumbone was washed and fresh medium was added to cells. Viability was assessed by XTT assay (Roche) after 48 h. The percent viability was calculated with respect to DMSO-treated controls. *Points*, mean of quadruplicates for each concentration; *bars,* SEM. (B, C) Clonogenicity: Cells were exposed to different concentrations of zerumbone for 4 h, and irradiated at respective doses of radiation. The drug was washed 3 h post-IR, and cells were trypsinized and replated in 6 well dishes in drug-free media. Cells were allowed to form colonies (8–14 days), which were then stained and counted. Results shown as means ± SEM of three independent experiments.

The stand-alone cytotoxicity of zerumbone on CRC cell lines was determined using XTT assay. Zerumbone inhibited the proliferation of all CRC cell lines tested (HCT116, HT29 and SW620) in a dose-dependent manner (Fig.1A). HCT116 cells were the most sensitive (IC_50_ 30 ± 1.5 *μ*mol/L), followed by SW620 (IC_50_ 38.8 ± 1.2 *μ*mol/L), whereas, HT29 cells were the most resistant to zerumbone treatment (IC_50_ > 46 *μ*mol/L).

### Zerumbone sensitized CRC cells to radiation

The effect of zerumbone on intrinsic tumor cell radiosensitivity of CRC cells was assessed by clonogenic cell survival assay. Zerumbone concentrations below IC_50_ were chosen for clonogenics (5, 10, and 25 *μ*mol/L). Treatment with zerumbone at concentrations of 10 and 25 *μ*mol/L significantly sensitized CRC cell lines HCT116 and HT29, whereas 5 *μ*mol/L zerumbone had no effect in improving the cellular radiosensitivity (Fig.1B and C). For HCT116 cells, the DEF at 0.1 SF were 1.01 ± 0.01 (5 *μ*mol/L zerumbone), 1.15 ± 0.04 (10 *μ*mol/L zerumbone), and 1.58 ± 0.03 (25 *μ*mol/L zerumbone), whereas, in HT29 cells, DEF values of 1 ± 0.01, 1.17 ± 0.08, and 1.46 ± 0.08 were calculated at 0.1 SF for 5, 10, and 25 *μ*mol/L of zerumbone, respectively.

### Zerumbone-enhanced radiation-induced cell cycle arrest and apoptosis

**Figure 2:**
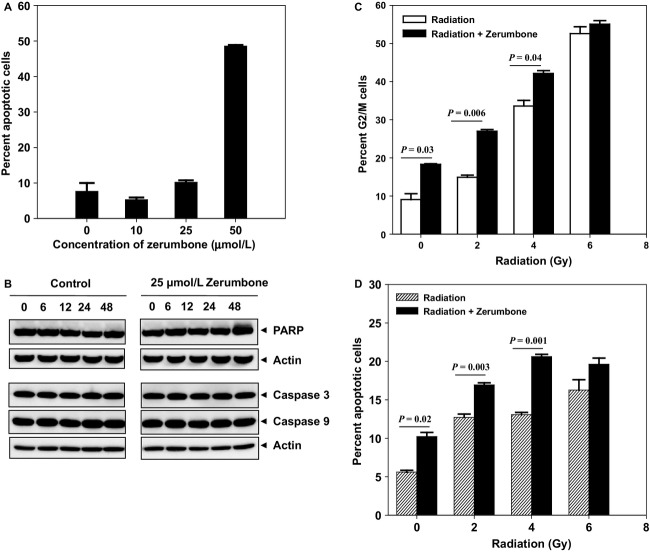
Zerumbone-induced apoptosis (with and without radiation): HCT116 cells were treated with zerumbone (4 h), irradiated, zerumbone removed 3 h post-IR and cells were incubated for further 48 h. (A) Percent apoptotic cells were estimated by PI staining using flow cytometry and (B) In a separate experiment, HCT116 cells were treated with zerumbone (25 *μ*mol/L), harvested at indicated time points and whole cell lysates were made for immunoblotting. (C) Zerumbone enhances radiation-induced G2/M arrest: Cells were treated with zerumbone (25 *μ*mol/L; 4 h), irradiated at different doses of radiation, zerumbone removed 3 h post-IR and cells further incubated. Cell cycle analysis done using PI staining at the end of 24 h post-IR (D) Zerumbone enhances radiation-induced apoptosis. HCT116 cells were treated with zerumbone (25 *μ*mol/L; 4 h), irradiated at different doses of radiation, zerumbone removed 3 h post-IR and percent apoptotic cells were estimated by PI staining using flow cytometry 48 h post-IR. *Columns*, Mean; *bars*, SEM. Representative data from one of the three independent experiments are shown.

To investigate if the effects of zerumbone-mediated radiosensitization were mediated through cell cycle arrest and/or apoptosis, we assessed the cell cycle distribution and apoptosis in HCT116 cells treated with zerumbone, with or without different doses of radiation by flow cytometry. Zerumbone alone induced dose-dependent apoptosis (Fig.2A). However, at radiosensitizing concentrations (10 and 25 *μ*mol/L), zerumbone-induced apoptosis was very low (5.3% and 10.2%, respectively) and was not significantly different than from untreated controls (*P *= 0.38; control vs. 10 *μ*mol/L and *P *= 0.34; control vs. 25 *μ*mol/L). We also treated HCT116 cells with 25 *μ*mol/L zerumbone, collected cell lysates at different time points (0, 6, 12, 24, and 48 h) and checked the expression of various markers of apoptosis (caspase 3, -9 and PARP). As seen in Figure2B, 25 *μ*mol/L zerumbone did not alter expression of any of the apoptotic proteins. When cells were treated with 25 *μ*mol/L zerumbone and radiation (Fig.2C), zerumbone not only arrested the cell cycle in G2/M itself (9% in untreated controls vs. 18.2% in zerumbone-treated; *P = *0.03), but also significantly enhanced radiation-induced G2/M arrest at 2 Gy (14.9% in radiation alone vs. 26.9% in radiation + zerumbone; *P = *0.006) and 4 Gy (33.6% in radiation alone vs. 42.1% in radiation + zerumbone; *P = *0.04). Though the G2/M arrest in the 6 Gy groups was not significantly different (*P = *0.3), the percent G2/M cells in zerumbone + radiation was slightly higher (55%) than 6 Gy alone group (52.6%). Similar trends were observed when cells were treated with zerumbone and radiation and analyzed for apoptosis 48 h post-IR (Fig.2D). Zerumbone alone induced 10.2% apoptosis (untreated controls showed 5.6% apoptosis; *P *= 0.02), and additionally, zerumbone treatment significantly enhanced the radiation-induced apoptosis at 2 and 4 Gy (12.7% and 13% in radiation alone vs. 16.9% and 20.6% in radiation + zerumbone; *P* = 0.003 and 0.001, respectively), though the difference between these two groups was not statistically significant at 6 Gy (*P = *0.2).

### Zerumbone prolonged postradiation DNA repair

**Figure 3:**
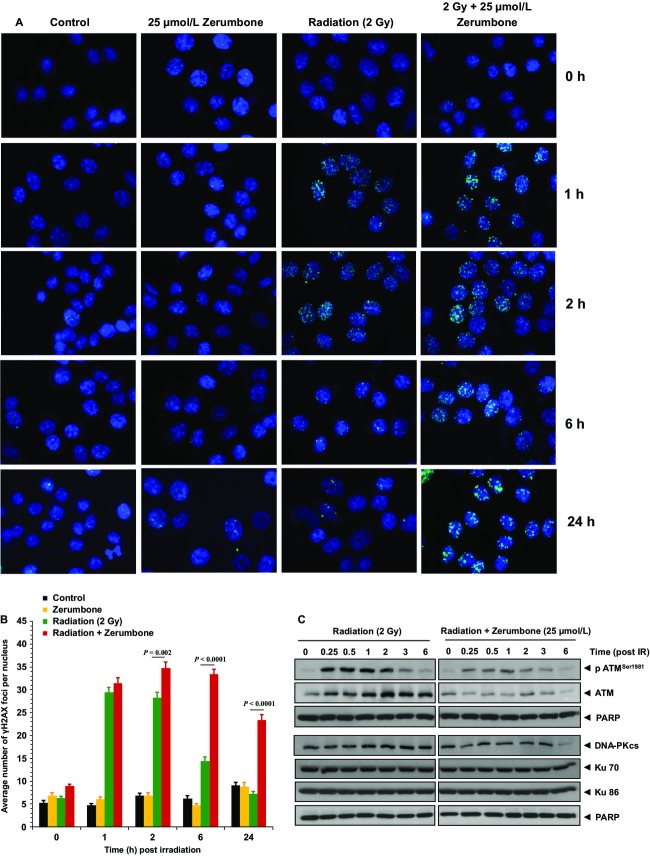
Zerumbone delays post-IR DNA repair. Zerumbone prolongs radiation-induced *γ*-H2AX foci. HCT116 cells were treated with 25 *μ*mol/L zerumbone (4 h), irradiated (2 Gy, zerumbone removed 3 h post-IR), and fixed at indicated time intervals for immunofluorescent staining of nuclear *γ*-H2AX foci. (A) Representative images for each treatment conditions; (B) Quantification of foci. *Columns *= Mean ± SEM of nuclear foci counted in 50 cells. (C) Effect of zerumbone on expression of DNA repair proteins. Cells were exposed to 25 *μ*mol/L zerumbone for 4 h and then irradiated (2 Gy, zerumbone removed 3 h post-IR). Cells were harvested at indicated time points post-IR, and nuclear fractions were subjected to immunoblotting. PARP used as loading control. Representative data from one of the three independent experiments are shown.

Repair of the damaged DNA following radiation is an important factor determining tumor radiosensitivity. To investigate if zerumbone could influence post-IR DNA repair, we assessed the expression of *γ*-H2AX foci as a measure of DNA double-strand breaks (DSBs). HCT116 cells were treated with zerumbone (25 *μ*mol/L), radiation (2 Gy), or both, and cells were processed for *γ*H2AX immunostaining at 1, 2, 6, and 24 h post-IR. Radiation alone induced the formation of *γ*H2AX foci within 1 h (29.4%), which gradually diminished to reach near baseline levels by 24 h post-IR (Fig.3A). In cells pretreated with zerumbone, the number of foci was slightly higher at 1 h post-IR (31.4%). However, the expression of foci continued to remain significantly higher than the radiation alone group at 2, 6, and 24 h post-IR (34.8, 33.4, and 23.3%, respectively; *P *< 0.005 for all groups). Interestingly, zerumbone treatment alone did not affect the baseline levels of foci at any time point (Fig.3A and B). We further assessed the effects of zerumbone with radiation on the nuclear expression of proteins involved in DDR such as ATM, DNA-PKcs, Ku70, and Ku86 in HCT116 cells. As seen in Figure3C, radiation induced the phosphorylation of ATM within 15 min post-IR, peaking at 30 min, and slowly diminishing around 3 h post-IR. Though treatment with zerumbone did not alter the post-IR phosphorylation kinetics of ATM, it decreased the overall expression levels of pATM^Ser1981^ at all these time points. Similar results were observed with DNA-PKcs. Following radiation, the nuclear expression of DNA-PKcs increased around 1 h post-IR and remained elevated till 6 h. Pretreatment with zerumbone decreased the radiation-induced expression of DNA-PKcs at all the time points, and almost completely abolished the DNA-PKcs expression at 6 h post-IR. However, neither radiation nor zerumbone + radiation treatment influenced the nuclear expression of Ku70 and Ku86.

### Zerumbone did not affect intracellular ROS production but depleted intracellular GSH

**Figure 4:**
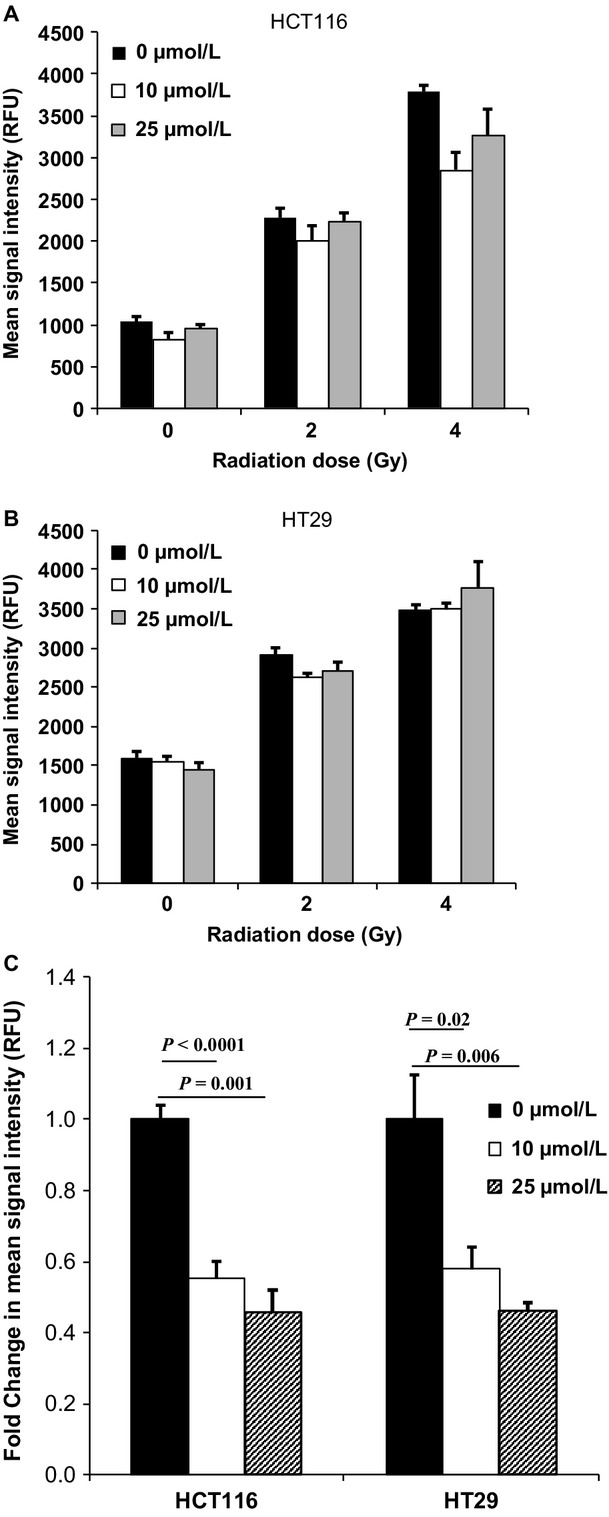
Zerumbone does not induce ROS but depletes cellular GSH. (A) HCT116 and (B) HT29 cells were seeded in 96 well plates and 48 h later were treated with indicated concentrations of zerumbone (*μ*mol/L) for 4 h, and then irradiated at indicated doses of radiation. Following 30 min post-IR incubation, the media was aspirated; cells were washed with PBS and incubated with CellROX® green reagent (Life Technologies) as per manufacturer's instructions. Cells were finally washed with PBS, and the fluorescence was measured using plate reader. *Columns*, Mean of triplicates, *bars*, SEM. *P > *0.05 (within groups with same radiation dose), hence not shown (C) Zerumbone depletes the intracellular GSH in both HCT116 and HT29 cells. Cells were plated in 96 well plates and 48 h later were treated with indicated concentrations of zerumbone (*μ*mol/L) for 4 h. The cells were then washed with PBS, and incubated with ThiolTracker™ Violet reagent (Life Technologies) according to manufacturer's instructions. Fluorescence was measured in a plate reader, and the fold change in mean fluorescence intensity was calculated with respect to untreated controls. Columns = Mean ± SEM of sextuplicates. Representative data of one of the three independent experiments are shown.

As zerumbone inhibited post-IR DNA repair and enhanced radiation-induced G2/M arrest, we checked if those effects were mediated through modulation of intracellular oxidative stress. Cells were treated with 10 and 25 *μ*mol/L zerumbone for 4 h, irradiated (2 and 4 Gy), and intracellular ROS generation was measured 30 min post-IR using CellROX® fluorogenic probes. In both HCT116 and HT29 cells, radiation-induced intracellular ROS, as seen by the increase in mean fluorescence signal intensity (in RFU). However, pretreatment with zerumbone neither influenced ROS generation by itself, nor did it enhance radiation-induced ROS generation at any radiation dose (Fig.4A). A one-way ANOVA showed that the differences between the groups (radiation; radiation + 10 *μ*mol/L zerumbone; radiation + 25 *μ*mol/L zerumbone) at any given radiation doses (2 or 4 Gy) were not statistically significant (*P *> 0.05), and zerumbone concentrations as high as 50 *μ*mol/L showed similar results (data not shown). We next checked if zerumbone affected the intracellular redox balance through alteration of cellular thiols. To estimate cellular GSH, cells were treated with zerumbone for 4 h and GSH levels were estimated using intracellular thiol probe ThiolTracker™ Violet at the end of the treatment. As seen in Figure4B, zerumbone treatment significantly depleted intracellular GSH in both cell lines. In HCT116 cells treated with 10 *μ*mol/L and 25 *μ*mol/L zerumbone, the GSH contents were reduced to 0.55- and 0.45-fold, respectively, compared to untreated controls (*P *< 0.0001; 0.001, respectively). Similarly, in HT29 cells, zerumbone treatment reduced the GSH content to 0.57 (10 *μ*mol/L) and 0.46 (25 *μ*mol/L) fold, significantly less than untreated controls (*P *= 0.02; 0.006, respectively).

### Pretreatment with only thiol-based antioxidants abolished zerumbone-mediated radiosensitization

**Figure 5:**
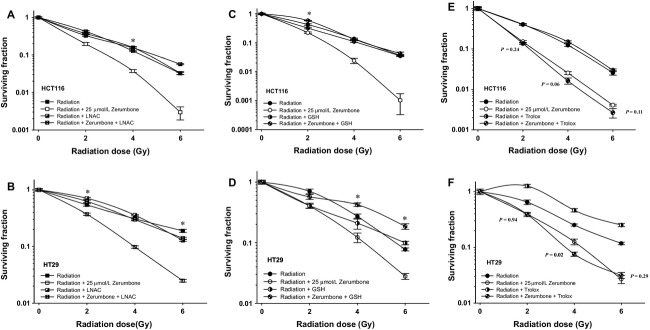
Only Thiol-based antioxidants abolish zerumbone-mediated radiosensitization. The clonogenic cell survival assays were repeated with HCT116 and HT29 and 25 *μ*mol/L of zerumbone in presence and absence of different antioxidants (A, B) 12 mmol/L *N*-acetyl-L cysteine for 24 h (C, D) 10 mmol/L GSH for 1 h and (E, F) 1 mmol/L Trolox (6-Hydroxy-2,5,7,8-tetramethylchromane-2-carboxylic acid; a water soluble form of vitamin E) for 24 h. Only LNAC and GSH, but not Trolox, completely reverted the radiosensitization effects of zerumbone in both these cells. Points =* *Mean of sextuplicates, bars = SEM. For (A–D), data were significantly different (*P *< 0.05) between radiation versus radiation + zerumbone + antioxidant only at points marked with an asterisk (*). For (E and F), statistical analysis done between radiation + zerumbone versus radiation + zerumbone + Trolox, and *P* value for each data point is indicated. Representative data from one of the three independent experiments are shown.

As zerumbone could deplete cellular GSH without enhancing ROS, we next observed the effects of thiol-based and nonthiol-based antioxidants on zerumbone-mediated radiosensitization. Cells were pretreated with NAC (12 mmol/L; 24 h) or GSH (10 mmol/L; 1 h) or Trolox (1 mmol/L; 24 h), and then were coexposed with 25 *μ*mol/L zerumbone for 7 h as indicated before. Pretreatment with both NAC and GSH completely abolished zerumbone-mediated radiosensitization in both HCT116 and HT29 cells (Fig.5A–D). The DEF (0.25 SF; compared with radiation only treatment controls) were as follows: HCT116 (5A), 1.72 (zerumbone) versus 1 (zerumbone + NAC); HCT116 (5C), 1.79 (zerumbone) versus 1.19 (zerumbone + GSH). For HT29 (5B), 1.86 (zerumbone) versus 1.06 (zerumbone + NAC); HT29 (5D), 1.58 (zerumbone) versus 0.81 (zerumbone + GSH). However, Trolox pretreatment did not revert zerumbone-mediated radiosensitization in either HCT116 (Fig.5E; DEF at 0.25 SF were 1.86 [zerumbone] versus 2.16 [zerumbone + Trolox]) or HT29 (Fig.5F; DEF at 0.25 SF were 1.49 [zerumbone] versus 1.85 [zerumbone + Trolox]). For Figures5A–D*,* The difference between the cell survival curves (radiation versus radiation + zerumbone + antioxidant) at each data set point (2, 4, or 6 Gy) was significantly different (*P < *0.05), as indicated in the figure. No significant difference between the groups at rest of the data points. For Figure5E–F, there was no significant difference between radiation + zerumbone versus radiation + zerumbone + Trolox at any of the data points (2, 4, and 6 Gy), as indicated by the (*P*) values in the figure. These results further affirmed that depletion of cellular GSH was the key mechanism behind zerumbone-mediated radiosensitization.

### The *α*,*β*-unsaturated carbonyl group is necessary for zerumbone-mediated radiosensitization

**Figure 6:**
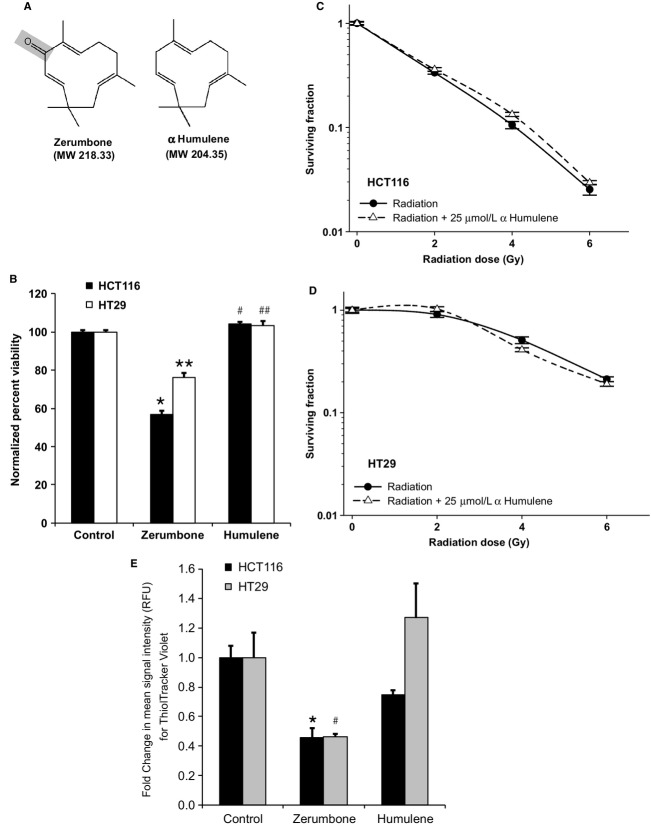
*α*,*β*-unsaturated carbonyl group is crucial in zerumbone-mediated radiosensitization. (A) Structure of zerumbone and *α* Humulene (HUM). HUM lacks *α*,*β*-unsaturated carbonyl group (gray). (B) HCT116 and HT29 cells were treated with zerumbone or HUM (25 *μ*mol/L) for 7 h and viability was determined 48 h later by XTT. Percent viability was normalized with respective untreated controls. HUM did not affect CRC cell viability at equimolar concentrations (*P < 0.0001, **P < 0.0001, #*P* = 0.02, ##*P* = 0.1 vs. respective controls). (C, D) Zerumbone did not sensitize HCT116 or HT29 cells toward radiation at equimolar (25 *μ*mol/L) concentrations. Points = Mean of sextuplicates, bars = SEM (E) HUM did not deplete the intracellular GSH levels in CRC cells, unlike zerumbone. Cells were treated with 25 *μ*mol/L of zerumbone or HUM for 4 h, and intracellular GSH contents were estimated using ThiolTracker™ Violet reagent (Life Technologies). Fold change in mean signal intensity was calculated using respective untreated controls. GSH depletion data of 25 *μ*mol/L zerumbone showed for comparison purpose. Columns = Mean of triplicates, bars = SEM. Representative data of one of the three independent experiments are shown (*P = 0.001, #*P* = 0.006 vs. respective controls).

The *α*,*β*-unsaturated carbonyl group is a key structural and chemical moiety of zerumbone (and other sesquiterpenes) and is considered essential for functional activity in many biological applications. We checked whether the *α*,*β* carbonyl group was essential for zerumbone-mediated radiosensitization. CRC cells were treated with HUM (25 *μ*mol/L), a zerumbone analog that lacks the *α*,*β* carbonyl group (Fig.6A) and cell viability and clonogenic assays (7 h treatment) were repeated. As seen in Figure6B, HUM did not show any stand-alone toxicity toward HCT116 and HT29 cells at 25 *μ*mol/L (viability in 25 *μ*mol/L zerumbone-treated cells was 56.8% [*P *< 0.0001 vs. control] and 76.1% [*P *< 0.0001 vs. control] for HCT116 and HT29, respectively), and the IC_50_ of HUM was >50 *μ*mol/L for both these cells (data not shown). Further, 25 *μ*mol/L HUM treatment did not sensitize either HCT116 or HT29 to radiation (Fig.6C–D; DEF at 0.1 SF = 0.93 and 1.01, respectively). Finally, HUM (25 *μ*mol/L) treatment failed to deplete intracellular GSH in both these cell lines (Fig.6E). The slight GSH decrease in HUM-treated HCT116 cells (0.74-fold) was not significantly different than untreated controls (*P* > 0.05). These results not only indicated that the *α*,*β*-unsaturated carbonyl group of zerumbone was the key for radiosensitization, but also reaffirmed that thiol depletion by zerumbone was a prerequisite for zerumbone-mediated radiosensitization in CRC cells.

## Discussion

In this study, we investigated whether sesquiterpene zerumbone from edible ginger could enhance the radiosensitivity of CRC cells in vitro. We first assessed the stand-alone toxicity of zerumbone in CRC cells and chose the radiosensitive, most sensitive to zerumbone HCT116 cells (wild-type p53; mutant *k-RAS*)^28^ and radioresistant, least sensitive to zerumbone HT29 cells (mutant p53; wild-type *k-RAS*)^28^ for further investigations. Although both cell lines were used to study the mechanism of zerumbone-mediated radiosensitization, the effect on cell cycle/apoptosis and DNA repair were studied in HCT116 cells, but not in HT29 cells for two reasons: (1) zerumbone treatment in HCT116 cells, but not in HT29 cells reduced the “shoulder” region of the radiation survival curve, which indicated inhibition of sub-lethal DNA damage repair as one of the prominent mechanism of action,^29^ and (2) “shoulderless” cell survival curves are also indicative of cells in late G2/M phase of the cell cycle.^30^ Zerumbone markedly inhibited the post-IR clonogenic survival of both CRC cells irrespective of their genetic framework (Fig.1), with comparable DEFs at 0.1 SFs. In HCT116 cells, zerumbone (25 *μ*mol/L) merely induced 10% apoptosis by itself, but significantly enhanced both radiation-induced cell cycle arrest in G2/M phase and radiation-induced apoptosis. Next, zerumbone significantly inhibited post-IR DNA repair (prolonged expression of nuclear *γ*H2AX foci) and decreased the nuclear expression of key DNA repair proteins, ATM and DNA-PKcs. Further, zerumbone-mediated radiosensitization was found to be stemming from its ability to deplete the cellular GSH pool, rather than enhancing ROS production (Fig.4) as only thiol-based antioxidants (NAC and GSH, but not Trolox) could completely abolish zerumbone-mediated radiosensitization in both HCT116 and HT29 cells (Fig.5). Finally, our results show that the *α*,*β*-unsaturated carbonyl group present in zerumbone was essential for its cytotoxic and radiosensitizing activities as zerumbone analog HUM, that lacks this functional group was nontoxic to CRC cells, had no radiosensitizing effects, and did not affect the cellular GSH pool at the same concentration (25 *μ*mol/L). These results also show that zerumbone's ability to deplete GSH was essential for its radiosensitizing potential.

Zerumbone have been known for its antiproliferative and anticancer effects in multiple cancer cell types,^22^ however, very little is known about its combination with other cancer therapeutic modalities (RT and chemotherapy). Particularly in CRC, zerumbone has been shown to have antiproliferative,^21^ anticarcinogenic,^23^ and proapoptotic^24^ effects, but whether zerumbone modulates the radiosensitivity of CRC cells is not known. The present study, therefore, is the first report of radiosensitizing effects of zerumbone in CRC.

Zerumbone treatment alone (and in combination with radiation) arrested cells in G2/M (Fig.2C), the most radiosensitive phase of the cell cycle. Agents that arrest the cell cycle in G2/M are often potent radiosensitizers,^31^,^32^ and zerumbone has been reported to trigger G2/M arrest in other cancer cell types.^33^ Cell cycle arrest in G2/M is primarily mediated through activation of checkpoints in response to the damaged DNA.^31^ Hence, we next checked the effect of zerumbone on IR-induced DNA damage.

Phosphorylation of the histone variant H2AX at Ser-139 residue (*γ*H2AX) is a rapid cellular response to DNA damage, which is also a well-established molecular marker to observe DNA damage initiation and resolution.^34^ We treated HCT116 cells with zerumbone and quantified the nuclear *γ*H2AX foci at multiple time points (0–24 h) post-IR. Zerumbone pretreatment increased the expression of radiation-induced *γ*H2AX foci at all these time points, and sustained the *γ*H2AX foci expression even at 24 h post-IR, where the *γ*H2AX foci in radiation alone group dropped to near baseline levels. Interestingly, zerumbone treatment alone did not induce *γ*H2AX foci formation. Prolonged expression of *γ*H2AX foci indicates a delayed DNA DSB repair. The plausible reason for such delay could be the altered expression of proteins involved in the DDR pathways. DNA DSBs are primarily repaired by either nonhomologous end joining (NHEJ) or homologous recombination (HR). These pathways involve sensor proteins that identify the DNA DSBs and in turn, activate proximal signal transduction kinases ATM and DNA-PKcs. Two of the identified canonical pathways are ATM activation through DSB-associated MRN (Mre11-Rad50-Nbs1)^35^ and Ku70 and DNA-PK-mediated NHEJ.^36^ Zerumbone pretreatment decreased the nuclear expression of radiation-induced pATM^Ser1981^, total ATM, and DNA-PKcs. However, the nuclear levels of Ku proteins were not affected. ATM is the central mobilizer of the cellular response to DNA DSBs,^37^ is crucial for HR pathway,^38^ and ATM inhibition has been known to translate into effective radiosensitization.^38^ Similarly, inhibitors of the enzyme DNA-PKcs have been shown to augment the tumor radiosensitivity.^39^,^40^ Hence, inhibition of both these key proteins by zerumbone is an attractive attribute to its radiosensitizing potential, and could be a possible mechanism underlying the delayed resolution of *γ*H2AX foci.

A plausible explanation for augmentation of radiation-induced DNA damage is enhanced ROS production. Hence, we next checked zerumbone's effects on intracellular ROS. Interestingly, zerumbone treatment (4 h) neither generated ROS by itself nor did it enhance radiation-induced ROS generation. Some sesquiterpene lactones (SLs) have been shown to exert their antitumor effects through ROS generation.^41^ On the other hand, zerumbone has been shown suppress free radical generation.^21^,^42^ Though some reports suggest zerumbone to induce ROS generation,^43^,^44^ this effect was observed only with a prolonged exposure to zerumbone (24 h) and at higher concentrations (>30 *μ*mol/L).

Sesquiterpene lactones possessing *α*,*β*-unsaturated carbonyl moiety show high reactivity with cellular thiols, resulting in alkylation of sulfhydryl groups of GSH through Michael-type addition reaction.^45^ This results in quick depletion of cellular GSH and protein thiols in cancer cells, resulting in disruption of cellular metabolism.^45^ We investigated if zerumbone-mediated radiosensitization involved GSH. Zerumbone treatment caused significant depletion of cellular GSH in both HCT116 and HT29 in just 4 h (Fig.4C), which indicated that like other SLs, zerumbone could not only quickly deplete cellular GSH, but this interaction was the key for zerumbone's bioactivity in CRC cells. To validate this hypothesis further, we performed clonogenic assays with different thiol-based (NAC and GSH) and nonthiol-based (Trolox) antioxidants. Pretreatment with only thiol-based antioxidants (NAC and GSH) could completely abrogate zerumbone-mediated radiosensitization, but not Trolox. This confirmed that in the 4 h treatment, the GSH-depleting ability of zerumbone was the prerequisite for its radiosensitizing properties. Lastly, HUM, the zerumbone analog lacking *α*,*β*-unsaturated carbonyl group was neither toxic nor could sensitize CRC cells to radiation. Most importantly, at same concentrations (25 *μ*mol/L), HUM could not deplete cellular GSH. Together, this indicated that the *α*,*β*-unsaturated carbonyl group of zerumbone and its interaction with cellular GSH was crucial for radiosensitization.

Glutathione depletion can disturb the cellular functions in multiple ways. Intracellular GSH depletion alone can induce apoptosis in cancer cells, independent of ROS generation.^46^,^47^ Additionally, GSH has recently been recognized to regulate important cellular functions beyond cellular redox balance such as DNA synthesis, gene expression, and repair of the radiation-induced DNA damage.^48^ Effects of GSH on radiation-induced DNA damage are complex, and recent studies indicate that in addition to protecting DNA from radiation-induced damage, GSH may also act as a modulator of DNA repair activity.^49^ In this regard, the ability of SLs to react with cellular thiols and thiol-bearing proteins is important for their antiproliferative effects as these can cause alterations in spatial structure and binding capability of proteins, causing disruption thiol-dependent and/or thiol-sensitive cellular protein activities that could be dependent or independent of each other.^45^

Our studies not only show for the first time that zerumbone could potently radiosensitize CRC cells, but also they elucidate an important aspect of zerumbone's ability to enhance tumor radiosensitivity through enhancing radiation-induced DNA damage. Most importantly, zerumbone-mediated radiosensitization was through a GSH-dependent mechanism that did not involve ROS.

Though this study doesn't address zerumbone's radiosensitizing effects on normal cells, SLs have been shown to selectively radiosensitize cancer cells, without affecting their normal counteparts.^41^ Zerumbone itself has shown selective toxicity toward colonic adenocarcinoma cells, with little effect on normal colon fiborblasts^21^ and owing to the intrinsic difference in the redox status of cancer cells and normal cells, it is proposed that zerumbone, at appropriate doses can be selectively toxic to cancer cells.^50^ In summary, though the detailed molecular mechanism(s) underlying zerumbone's effects on post-IR DNA damage remain to be elucidated, the current findings clearly suggest that continued evaluation of zerumbone as potential radiosensitizer is warranted as a segue to eventual clinical evaluation.
